# A Unique Surgical Case of Mixed Metaplastic Breast Carcinoma With Heterologous Mesenchymal Differentiation and Conventional Adenocarcinomatous Elements

**DOI:** 10.7759/cureus.55926

**Published:** 2024-03-11

**Authors:** Yoshiiku Okanemasa, Akihiro Shioya, Motona Kumagai, Mao Takata, Yumi Tsubata, Jia Han, Toshie Terauchi, Emi Morioka, Masafumi Inokuchi, Sohsuke Yamada

**Affiliations:** 1 Department of Pathology, Kanazawa Medical University Hospital, Kahoku, JPN; 2 Department of Pathology and Laboratory Medicine, Kanazawa Medical University, Uchinada, JPN; 3 Department of Pathology II, Kanazawa Medical University, Kahoku, JPN; 4 Department of Pathology and Laboratory Medicine, Kanazawa Medical University, Kahoku, JPN; 5 Department of Breast and Endocrine Surgery, Breast Center, Kanazawa Medical University Hospital, Kahoku, JPN; 6 Department of Pathology and Laboratory Medicine, Kanazawa Medical University, Ishikawa, JPN

**Keywords:** cytopathology, needle aspiration, heterologous mesenchymal differentiation, invasive, metaplastic breast carcinoma (mbc)

## Abstract

Metaplastic breast carcinoma (MBC) is very rare among all invasive breast carcinomas, accounting for less than 1.0% of them. MBCs are classified into five subtypes, including mixed MBC - where the mix might be multiple metaplastic elements or a mixture of epithelial and mesenchymal elements. Overall survival for mixed MBC tends to correlate with a significantly worse outcome. Therefore, an early accurate diagnosis and surgical treatment for mixed MBCs must allow for an improved quality of life and better prognosis. However, there have not been many recently published papers describing the detailed cytological features of mixed MBCs on fine-needle aspiration (FNA) specimens.

A 60-year-old female presented with a history of a hard breast mass on the left lateral side, showing an ill-defined and marginally enhanced tumor nodule on magnetic resonance imaging. The cytologic specimens of FNA contained a large number of three-dimensional, cohesive and sheet-like clusters, or non-cohesive single cells, of highly atypical spindled sarcomatoid to oval epithelioid cells having hyperchromatic pleomorphic nuclei and mitotic figures, in a necrotic and hemorrhagic background. A small amount of osteoid matrix-like substance was rarely seen, associated with a very small number of osteoclast-like giant cells. We first interpreted it as an invasive breast carcinoma of high grade. A mastectomy was performed, and a gross examination of the neoplasm revealed a hemorrhagic solid tumor lesion with a gray-whitish cut surface, measuring approximately 35 × 24 × 21 mm in diameter. On a microscopic examination, the tumor was predominantly composed of the proliferation of highly atypical oval to spindled cells predominantly in a sarcomatous growth fashion with focal production of chondroid and osteoid matrix, peripherally coexisted with a smaller volume of conventional invasive breast carcinoma. Immunohistochemistry showed that the sarcomatous tumor cells were specifically positive for vimentin, α-smooth muscle actin, or epithelial membrane antigen. Therefore, we finally made a diagnosis of invasive mixed MBC with heterologous mesenchymal differentiation and conventional adenocarcinomatous elements.

To the best of our knowledge, this would most recently be the first case report of mixed MBC with heterologous mesenchymal differentiation and conventional adenocarcinomatous elements, with a focus on its FNA cytomorphologic findings. We should be aware that owing to its characteristic cytological features, cytopathologists might be able to make a correct diagnosis of MBC, based on multiple and adequate samplings.

## Introduction

Metaplastic breast carcinoma (MBC) is very rare among all invasive breast carcinomas, accounting for less than 1.0% of them [1,2]. The current WHO Tumour Classification of the Breast 5th Edition has maintained a descriptive morphological classification system based on the characteristically metaplastic elements [1]. Actually, MBCs are classified into (i) low- to high-grade adenosquamous carcinomas, (ii) pure squamous cell carcinoma, (iii) pure spindle cell carcinoma, (iv) fibromatosis-like MBC, (v) MBC with heterologous mesenchymal differentiation, including chondroid (myxoid/cartilaginous), osseous (bone), rhabdomyoid (muscle) and neuroglial components, and (vi) mixed MBC - where the mix might be multiple metaplastic elements or a mixture of epithelial and mesenchymal elements [1,2]. By contrast, to date, their sufficient and valuable molecular data still remain to be elucidated, despite the fact that most of the MBCs are typically triple-negative (lacking expressions of estrogen receptor (ER), progesterone receptor (PgR) and HER2/ErbB2) [2].

MBC often poses a diagnostic challenge to all clinicians and cytopathologists, since its highly heterogeneous entity should be very difficult to diagnose pre-operatively, especially on small, inadequate samples. Indeed, a number of MBC cases might result in paucicellular cytological specimens with many markedly degenerative and poorly preserved (i.e., non-viable) carcinoma cells [3-5]. Furthermore, overall survival for several types of MBCs, including mixed MBCs, tends to correlate with a significantly worse outcome, occasionally displaying distant metastases of the brain and/or lungs [1,2]. Therefore, an early accurate diagnosis and surgical treatment for the MBCs must allow for an improved quality of life and better prognosis. However, there have not been many recently published papers describing the detailed cytological features of MBCs on fine-needle aspiration (FNA) specimens.

To the best of our knowledge, this would most recently be the first case report of mixed MBC with heterologous mesenchymal differentiation and conventional adenocarcinomatous elements, with a focus on its FNA cytomorphologic findings. In that sense, the present brief case report potentially interests the scientific community.

## Case presentation

A 60-year-old female patient with unremarkable previous medical and family histories incidentally had a chief complaint of a relatively hard left breast mass of the lateral side (CD-portion). Breast magnetic resonance imaging (MRI) showed an ill-defined and marginally enhanced tumor nodule with internal, focal low-density areas, measuring approximately 25 × 23 × 21 mm in diameter, arising from the upper middle quadrants of the left breast. Full-body CT revealed no definite evidence of metastases or neoplastic foci in the lymph nodes or other organs.

**Figure 1:**
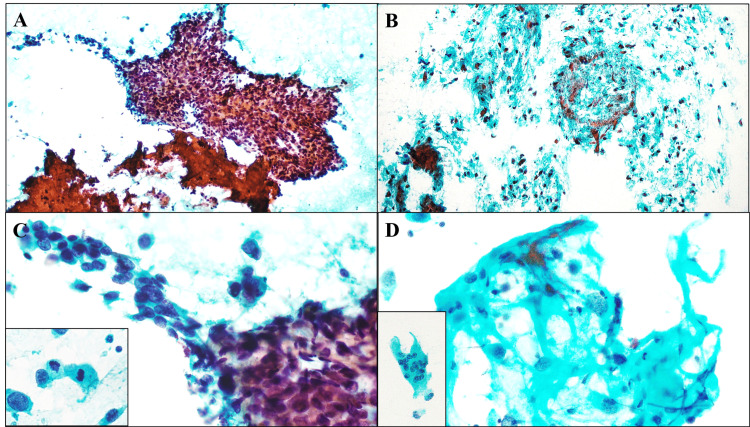
The findings of FNA cytologic/cytomorphologic examination on the present mixed MBC with heterologous mesenchymal differentiation and conventional adenocarcinomatous elements. A) The FNA cytology specimen from the tumor nodule (Papanicolaou staining) contains a large number of small to large, cohesive sheet-like and three-dimensional clusters of viable and highly atypical spindled to oval epithelioid cells with loss of myoepithelial components, in a necrotic and hemorrhagic background. Original magnification: 100×. B) Moreover, in other areas, a large number of non-cohesive single cells of viable and highly atypical spindled sarcomatoid cells are noted. Within our thorough observation, there are no clusters of apparent conventional adenocarcinoma cells. Original magnification: 100×. C) On a high-power view (Papanicolaou staining), those tumor cells reveal hyperchromatic pleomorphic nuclei and prominent nucleoli, admixed with a number of mitotic figures (inset), displaying poorly differentiated features. Original magnification: 400× (inset 400×). D) A small amount of characteristic osteoid matrix-like substance is rarely seen, associated with a very small number of osteoclast-like giant cells (inset). Original magnification: 400× (inset 400×). FNA: fine-needle aspiration; MBC: metaplastic breast carcinoma

The specimen of the FNA cytology sample from the tumor nodule unexpectedly contained a large number of small to large, cohesive sheet-like and three-dimensional clusters, or non-cohesive single cells, of viable and highly atypical spindled sarcomatoid to oval epithelioid cells with loss of myoepithelial components, in a necrotic and hemorrhagic background on Papanicolaou stain (Figures 1A-1B). Those tumor cells had hyperchromatic pleomorphic nuclei and prominent nucleoli admixed with a number of mitotic figures, displaying poorly differentiated features (Figure 1C). Within our thorough observation, a small amount of osteoid matrix-like substance was rarely seen, associated with a very small number of osteoclast-like giant cells (Figure 1D). No apparent squamoid/squamous carcinoma cells were identified. In addition, there were no clusters of apparent conventional adenocarcinoma cells. Giemsa staining was not performed. We first interpreted these pictures as indicating malignancy, highly suggestive of an invasive breast carcinoma of high grade, but could not completely exclude the possibilities of malignant phyllodes tumor or primary breast sarcoma, such as chondrosarcoma or undifferentiated pleomorphic sarcoma.

**Figure 2:**
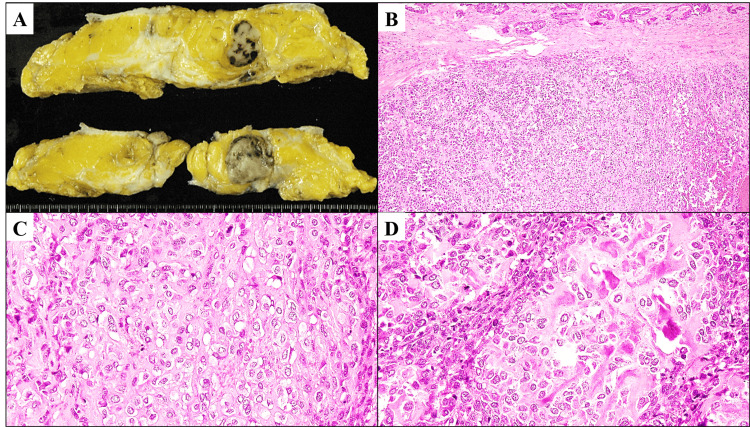
The gross and histopathological findings on the present mixed MBC with heterologous mesenchymal differentiation and conventional adenocarcinomatous elements. A) The breast tumor grossly reveals a solid and poorly demarcated mass lesion with a gray-whitish cut surface, associated focally with hemorrhage and fat invasion, measuring approximately 35 × 24 × 21 mm area in diameter. B) On a microscopic low-power view (H&E staining; ×100), its solid parts are predominantly composed of the proliferation of highly atypical cells with absence of two-cell patterns in a sarcomatous growth fashion (middle to bottom), peripherally coexisted with a small volume of conventional invasive breast carcinoma in an irregular tubular and trabecular pattern (the most upper). These tumor nests aggressively invade the surrounding stroma/fat with focal desmoplastic fibrosis. C,D) A higher power view (H&E staining; ×400) reveals that these neoplastic cells have hyperchromatic and pleomorphic, oval to spindled nuclei, conspicuous nucleoli, and numerous mitotic figures, with focal chondroid (C) and osteoid (D) differentiation, accompanied by production of chondroid (C) and osteoid (D) matrix. MBC: metaplastic breast carcinoma

Mastectomy was thus performed, and a gross examination of the tumor revealed a solid and poorly demarcated mass lesion with a gray-whitish cut surface, associated focally with hemorrhage and fat invasion, measuring approximately 35 × 24 × 21 mm area in diameter (Figure 2A). Resection was deemed to be complete by this histopathological examination. Microscopically, the solid parts of the tumor were predominantly composed of the proliferation of highly atypical cells with absence of two-cell patterns in a sarcomatous growth fashion, peripherally coexisted with a small volume of conventional invasive breast carcinoma in an irregular tubular and trabecular pattern (Figure 2B). On a high-power view, these neoplastic cells had hyperchromatic and pleomorphic, oval to spindled nuclei, conspicuous nucleoli, and numerous mitotic figures, with focal chondroid and osteoid differentiation, accompanied by production of chondroid and osteoid matrix (Figures 2C-2D). Furthermore, these tumor nests aggressively invaded the surrounding fat with focal desmoplastic fibrosis (Figure 2B).

**Figure 3:**
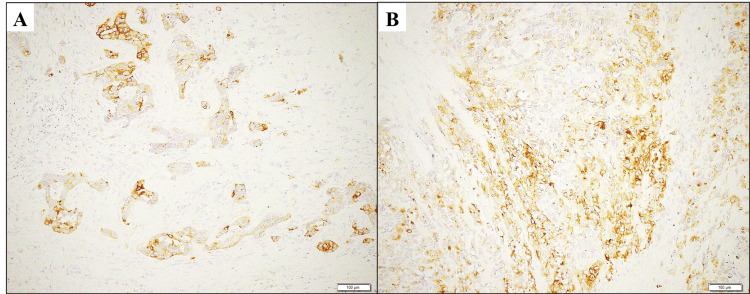
The immunohistochemical (EMA) findings on the present mixed MBC with heterologous mesenchymal differentiation and conventional adenocarcinomatous elements. (A,B) The immunohistochemical findings (×200) demonstrate that both of the adenocarcinomatous (A) and sarcomatous (B) tumor cells are specifically positive for epithelial membrane antigen (EMA). MBC: metaplastic breast carcinoma

Immunohistochemical findings showed that both of the sarcomatous and adenocarcinomatous tumor cells were completely negative for ER/PgR/HER2 (i.e., triple-negative), p40, p63, S-100 protein, myogenin/MyoD1 and CD34, whereas they were uniquely and specifically positive for epithelial membrane antigen (EMA) (Figures 3A-3B). Moreover, the conventional adenocarcinoma cells were specifically positive for cytokeratins and E-cadherin. While the sarcomatous tumor cells were specifically positive for vimentin and α-smooth muscle actin. The Ki67 (MIB-1) labeling index was very high, approximately up to 78%.

Based on these features, we ultimately made a diagnosis of mixed MBC with heterologous mesenchymal differentiation (including chondroid and osseous components) and conventional adenocarcinomatous elements. Neither lymph nodes metastasis nor distant metastasis was not observed. To date, we have completed approximately 2 years of routine follow-up since the surgery, and the patient remains well without any definite evidence of recurrences or metastases.

## Discussion

The cytological features of MBCs can more or less reflect the heterogeneously histopathological ones, and it must be easy for cytopathologists to be diagnosed conclusively as malignant in most of them [3-6]. However, the cytologically correct diagnosis of MBCs might remain problematic in all of those cases, because of inadequate and/or selective sampling of significantly various elements [3,6]. FNA of MBCs could cytologically show small to large, cohesive to non-cohesive, three-dimensional clusters or single cells of diverse tumor components, such as sarcomatoid malignant cells, poorly differentiated carcinoma cells, squamous carcinoma cells or conventional adenocarcinoma cells [1,3-5]. These tumor clusters and/or single cells often display the presence of apparent nuclear hyperchromatism and pleomorphism, prominent nucleoli, or mitotic figures, with or without chondroid or osteoid or myxoid matrix [3-5]. According to the previous series of FNA cytology findings on MBCs [3,5], the identification of dual elements, such as unequivocal squamous carcinoma cells and chondroid/osteoid stroma, is very helpful for accurate diagnosis of MBC. Moreover, the presence of poorly differentiated, atypical spindle cells and/or osteoclast-like giant cells could strongly give rise to the suggestion of MBC [3]. In this context, as in our case, since the specimens were fully adequate, the cytologic features were mostly similar to those of MBC, as described above, even though no apparent squamous/squamoid carcinoma cells were observed. Furthermore, we can specifically show the correspondence between cytological and histological findings, except for no cytological clusters of apparent conventional adenocarcinoma cells. Thus, a conclusive and accurate diagnosis of MBC, based on cytology alone, might be possible to achieve. Nevertheless, in any cases with malignant breast tumors, multiple rounds of ultrasound-guided (if possible) FNA cytology are recommended [7].

Finally, with regard to this case of mixed MBC with heterologous mesenchymal differentiation and conventional adenocarcinomatous elements, malignant phyllodes tumor, primary chondrosarcoma or primary undifferentiated pleomorphic sarcoma can be included in the differential diagnoses. Those cytologic features are considered to reveal complete absence of highly atypical epithelial-like cells forming cohesive sheet-like and “three-dimensional” clusters [4]. Furthermore, the findings of the current immunohistochemical analyses suggest that immunostaining for specific epithelial markers, such as EMA, on sarcomatoid elements in cytological smears (i.e., immunocytochemistry) must be useful for the differential diagnosis with malignant phyllodes tumor or primary breast sarcomas [7-9].

## Conclusions

We should be aware that owing to its characteristic cytological features, cytopathologists might be able to make a correct diagnosis of MBC, based on multiple and adequate samplings. Initially, accurate diagnosis with cytology could lead to neoadjuvant chemotherapy for MBC, as one of the benefits, however, its effectiveness has not seemingly been very good. To the best of our knowledge, this would most recently be the first case report of mixed MBC with heterologous mesenchymal differentiation and conventional adenocarcinomatous elements, with a focus on its FNA cytomorphologic findings. In that sense, the present brief case report potentially interests the scientific community.
